# Unbiased chromatin accessibility profiling by RED-seq uncovers unique features of nucleosome variants in vivo

**DOI:** 10.1186/1471-2164-15-1104

**Published:** 2014-12-15

**Authors:** Poshen B Chen, Lihua J Zhu, Sarah J Hainer, Kurtis N McCannell, Thomas G Fazzio

**Affiliations:** Program in Gene Function and Expression, University of Massachusetts Medical School, Worcester, MA 01605 USA; Program in Molecular Medicine, University of Massachusetts Medical School, Worcester, MA 01605 USA; Program in Bioinformatics and Integrative Biology, University of Massachusetts Medical School, Worcester, MA 01605 USA

**Keywords:** RED-seq, Restriction enzyme accessibility, Chromatin accessibility, Nucleosome dynamics, Embryonic stem cells

## Abstract

**Background:**

Differential accessibility of DNA to nuclear proteins underlies the regulation of numerous cellular processes. Although DNA accessibility is primarily determined by the presence or absence of nucleosomes, differences in nucleosome composition or dynamics may also regulate accessibility. Methods for mapping nucleosome positions and occupancies genome-wide (MNase-seq) have uncovered the nucleosome landscapes of many different cell types and organisms. Conversely, methods specialized for the detection of large nucleosome-free regions of chromatin (DNase-seq, FAIRE-seq) have uncovered numerous gene regulatory elements. However, these methods are less successful in measuring the accessibility of DNA sequences within nucelosome arrays.

**Results:**

Here we probe the genome-wide accessibility of multiple cell types in an unbiased manner using restriction endonuclease digestion of chromatin coupled to deep sequencing (RED-seq). Using this method, we identified differences in chromatin accessibility between populations of cells, not only in nucleosome-depleted regions of the genome (e.g., enhancers and promoters), but also within the majority of the genome that is packaged into nucleosome arrays. Furthermore, we identified both large differences in chromatin accessibility in distinct cell lineages and subtle but significant changes during differentiation of mouse embryonic stem cells (ESCs). Most significantly, using RED-seq, we identified differences in accessibility among nucleosomes harboring well-studied histone variants, and show that these differences depend on factors required for their deposition.

**Conclusions:**

Using an unbiased method to probe chromatin accessibility genome-wide, we uncover unique features of chromatin structure that are not observed using more widely-utilized methods. We demonstrate that different types of nucleosomes within mammalian cells exhibit different degrees of accessibility. These findings provide significant insight into the regulation of DNA accessibility.

**Electronic supplementary material:**

The online version of this article (doi:10.1186/1471-2164-15-1104) contains supplementary material, which is available to authorized users.

## Background

Eukaryotic genomes are wrapped around histone octamers to form nucleosome arrays, which are further packaged into the nucleus. Although chromatin compaction facilitates storage of large quantities of DNA within small nuclear compartments, it drastically reduces the accessibility of genomic DNA to proteins that require access. Nucleosomal DNA is relatively inaccessible to DNA binding proteins due to both the occlusion of approximately half of its surface by contacts with histones, as well as the distortion of the normal B-form structure that occurs when DNA is wrapped around a histone octamer [1]. Consequently, chromatin structure must be disrupted to facilitate normal cellular processes, such as DNA repair, recombination, replication, and transcription.

Although protection of DNA from nuclear factors by the formation of tight interactions with histones appears to be the major method by which DNA accessibility is regulated, many different isoforms of the histone octamer exist within most eukaryotes, each with distinct biochemical and biophysical properties [2–8]. These differences are mainly derived from two sources. First, most eukaryotes express several variants each of histones H2A and H3. Within each family, differences between variants can range from a few amino acid substitutions to the presence or absence of additional, non-histone domains at their amino- or carboxyl-termini. Second, all four core histone proteins are subject to a wide array of post-translational modifications, including acetylation, methylation, phosphorylation, ubiquitylation, and others. Several of these modifications and variants change the overall charge of the histone octamer and/or create or destroy binding sites for proteins, resulting in alterations in nucleosome stability [5, 9–11]. Together, these differences in nucleosome structure and stability conferred by histone variants and modifications raise the possibility that accessibility of nucleosomal DNA may not be a simple binary phenomenon in which nucleosome-bound DNA is completely protected and nucleosome-free DNA is completely accessible; rather, DNA within some variants of nucleosomes may be more accessible than DNA bound by other variants. For example, nucleosomes harboring histone variants H2A.Z and/or H3.3 are extractable from bulk chromatin at lower salt and, in some cases, protect smaller footprints of DNA from nucleases than canonical nucleosomes [6, 12–14], raising the possibility that DNA within certain nucleosome variants is more broadly accessible, due to either biophysical properties or dynamic behavior of these nucleosomes. However, this possibility remains to be directly tested *in vivo*.

Along with differences in chromatin structure within distinct genomic regions in individual cell types, cell type-specific chromatin structural differences facilitate gene expression patterns specific to cells of different lineages [15]. In embryonic stem cells (ESCs), chromatin structure is relatively open (less heterochromatic) compared to differentiated cells, which may be necessary for their ability to self-renew (proliferate as ESCs) while maintaining the flexibility to turn on lineage-specific genes during differentiation [16, 17]. As ESCs differentiate, DNA accessibility decreases, chromatin becomes less dynamic, and larger blocks of heterochromatin form, suggesting that differentiation induced chromatin alterations may stabilize cell fates by “locking down” regions of the genome in heterochromatic blocks that are relatively insensitive to transcriptional activators.

Methods have been developed to study DNA accessibility based on either the protection of nucleosomal DNA from general endonuclease digestion or the differential solubility properties of open and closed chromatin. Deoxyribonuclease I (DNase I) [18, 19] preferentially digests nucleosome-free DNA [20–22], and genomic regions that are more sensitive to DNase I digestion – called DNase I hypersensitive sites (DHSs) – can be identified by deep sequencing (DNase-seq) [23]. Formaldehyde-Assisted Isolation of Regulatory Elements (FAIRE) is a second method to isolate accessible genomic regions, which uses organic extractions of formaldehyde cross-linked chromatin to enrich protein-free DNA fragments that are subsequently identified by microarrays (FAIRE-chip) [24] or high-throughput sequencing (FAIRE-seq) [25]. Consistent with the requirement of most transcription factors (TFs) for accessible binding sites on DNA, DHSs and FAIRE-seq peaks are enriched for regulatory regions of active genes (enhancers and promoters). Conversely, micrococcal nuclease digestion of chromatin followed by deep sequencing of the regions of DNA protected from digestion (MNase-seq) allows inference of the positions and occupancy levels of nucleosomes in a population (when footprints of ~150 bp are quantified) and TFs (when footprints less than ~80 bp are considered) [22, 26–28]. When compared to maps of nucleosome positions, both DNase-seq and FAIRE-seq tend to identify large nucleosome-depleted regions that range from 100-300 bp in length [29]. As a result, differences in DNA accessibility that occur within or close to nucleosomes, or quantitative differences in accessibility of individual nucleosomes, are difficult to detect by these methods.

In addition, for more than three decades, restriction enzymes (REs) have been utilized to probe DNA accessibility at individual loci [30–34]. Since REs digest DNA at specific nucleotide sequences known as restriction sites (RSs), REs can quantitatively probe cell type-specific differences in accessibility at individual positions, when combined with Southern blotting or PCR. The accessibility of chromatin to REs can, in principle, be quantified at any genomic location that harbors an RS, including DHSs, DNA sequences within nucleosomes, and linker regions within closely-spaced nucleosome arrays. Previously, Gargiulo *et al.* developed a genome-wide method to probe chromatin structure using restriction enzymes, finding that chromatin accessibility correlated broadly with gene expression in hematopoietic cell lineages and became progressively restricted during differentiation [35]. Here we modified this method to reduce potential biases in library production and increase the fraction of reads within a library that directly reflect RE cleavage. We employ this modified method, termed RED-seq, to measure RE accessibility across the genome of multiple cell types.

Here we show that, as with DNase-seq and FAIRE-seq, RED-seq uncovers known regions of open chromatin, validating the method as a genome-wide probe of chromatin accessibility. Furthermore, we find that RED-seq can quantify both large differences in chromatin accessibility between different cell types and subtle changes that occur during ESC differentiation, highlighting the sensitivity of the assay. However, unlike these methods, we find that RED-seq also identifies differences in accessibility within nucleosome arrays. Consequently, we uncover significant differences in accessibility between nucleosomes containing different histone variants, showing that DNA bound by nucleosomes containing H2A.Z or H3.3 are more accessible than the genome-wide average. Consistent with this model, RNAi-mediated depletion of factors required for H2A.Z or H3.3 deposition into chromatin results in reduction of accessibility at these sites. Therefore, these results provide *in vivo* evidence that DNA accessibility within nucleosomes is modulated by the composition of histone proteins.

## Results

### Genome-wide measurement of chromatin accessibility by RED-seq

Due to the inherent biases of standard methods of measuring chromatin accessibility, such as DNase-seq and FAIRE-seq, toward nucleosome-free regions of DNA, these methods are not well suited to examination of chromatin accessibility in the vast majority of the genome found within nucleosome arrays. A prior RE-based method of probing chromatin accessibility genome-wide (called NA-Seq) revealed that accessibility of regulatory regions of genes correlated with their gene expression patterns [35]. We therefore wished to examine the accessibility of ESC chromatin using REs, in order to probe regions of open chromatin structure that are well covered by DNase-seq and FAIRE-seq maps (to assess whether REs faithfully report known features of ESC chromatin structure), as well as examine chromatin accessibility within nucleosomes and between nucleosomes that lie within regularly-spaced nucleosome arrays.

**Figure 1:**
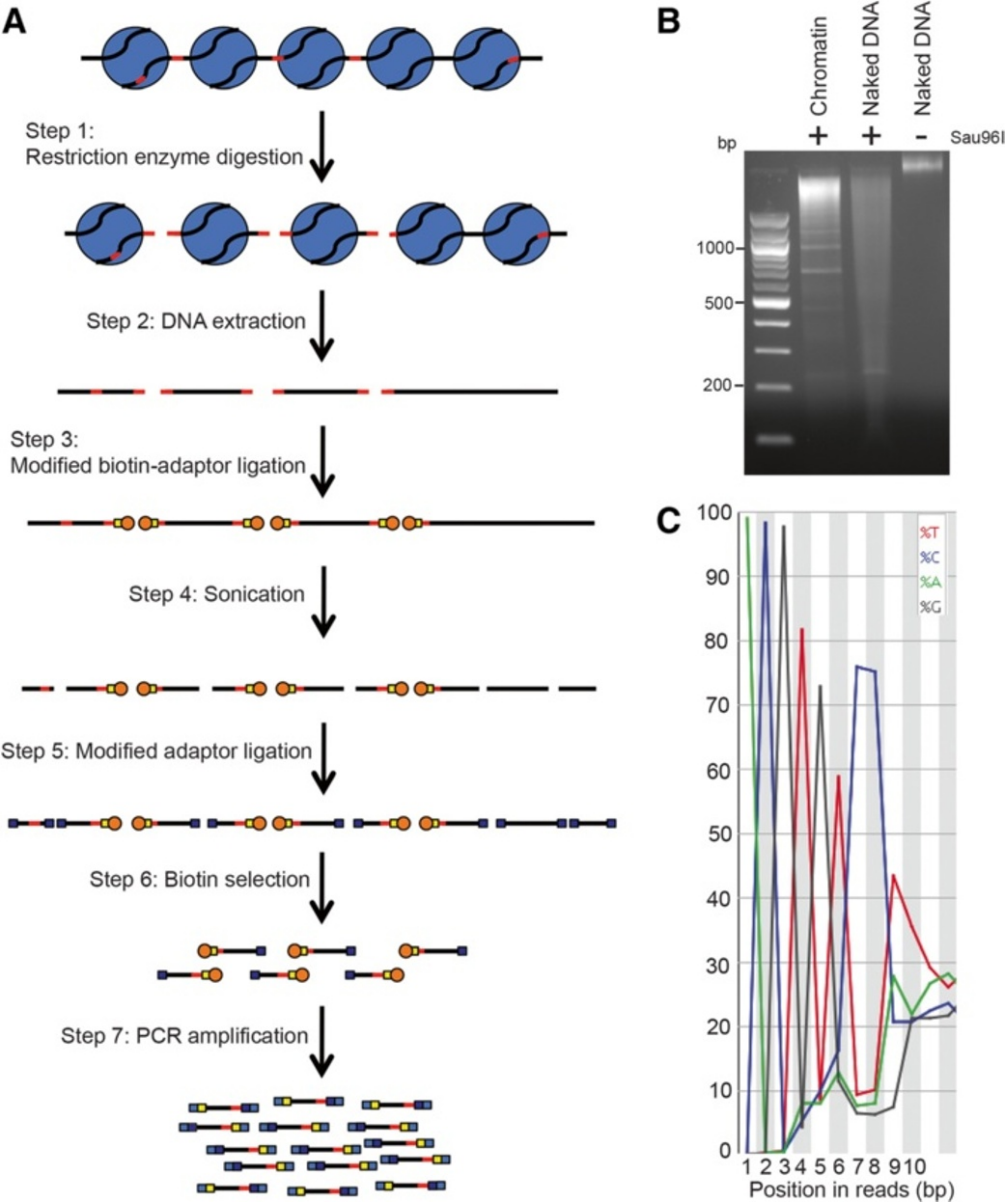
**The RED-seq method for genome-wide measurement of RE accessibility. (A)** RED-seq workflow. RSs are shown in red, yellow boxes (Step 3) represent RS-proximal adaptors, dark blue boxes (Step 5) represent RS-distal adaptors, orange circles represent biotin, light blue boxes represent paired-end PCR primers, large blue circles (Step 1) represent nucleosomes, and DNA is shown in black. **(B)** Ethidium bromide stained agarose gel indicating bulk digestion levels of chromatin and naked DNA. **(C)** An example FASTQ file is shown to illustrate the near-uniform sequencing of the RS-containing end of each fragment in the library, signified by the large enrichment of G at position 5, and a CC dinucleotide at positions 7 and 8, derived from the cleaved and blunt-ended Sau96I site (GNCC).

NA-Seq was previously performed by exposing purified nuclei to REs, secondary digestion of the purified DNA with an additional RE, ligation of linkers, and 454 pyrosequencing [35]. We modified the NA-Seq method in several ways (Figure 1A): First, we performed RE digestion on permeabilized cells without nuclear purification in order to reduce processing steps prior to chromatin digestion by REs. Second, we used an unbiased, sonication-based shearing approach after DNA purification to reduce potential biases in the library introduced by the genomic distribution of the restriction sites (RSs) specific for the post-DNA purification RE used in NA-Seq. Finally, we used two separate linker ligation steps to ensure that single-read Illumina sequencing would sequence the end of each DNA fragment cleaved by the RE (rather than the randomly sheared end), making nearly all mapped reads informative, rather than about half. We refer to this modified method as RED-seq to distinguish this modified protocol from the previous NA-Seq approach.

In principle, any RE or combination of REs could be used for RED-seq library preparation. We utilized Sau96I, an RE with a four base RS (GGNCC) that occurs frequently throughout the mouse genome and is abundant within gene regulatory sequences, in order to probe genome-wide accessibility at relatively high resolution. First, we compared the differences in RE accessibility between mouse ESC chromatin and naked DNA. Because chromatin and naked DNA have identical RSs, differences in RE accessibility should result directly from the influences of chromatin proteins on accessibility at each RS (e.g., nucleosome occupancy or binding of non-histone proteins). Indeed, naked DNA was more efficiently cleaved and the digestion products were more uniformly distributed compared to ESC chromatin (Figure 1B), as expected. Next, we prepared sequencing libraries of ESC and naked DNA samples, to quantify the digestion frequency at each Sau96I RS in the genome, and sequenced the libraries. The enrichment within the sequence reads of the expected product of Sau96I digestion (GNCC) immediately following the adapter barcode confirmed the quality of the libraries (Figure 1C).

**Figure 2:**
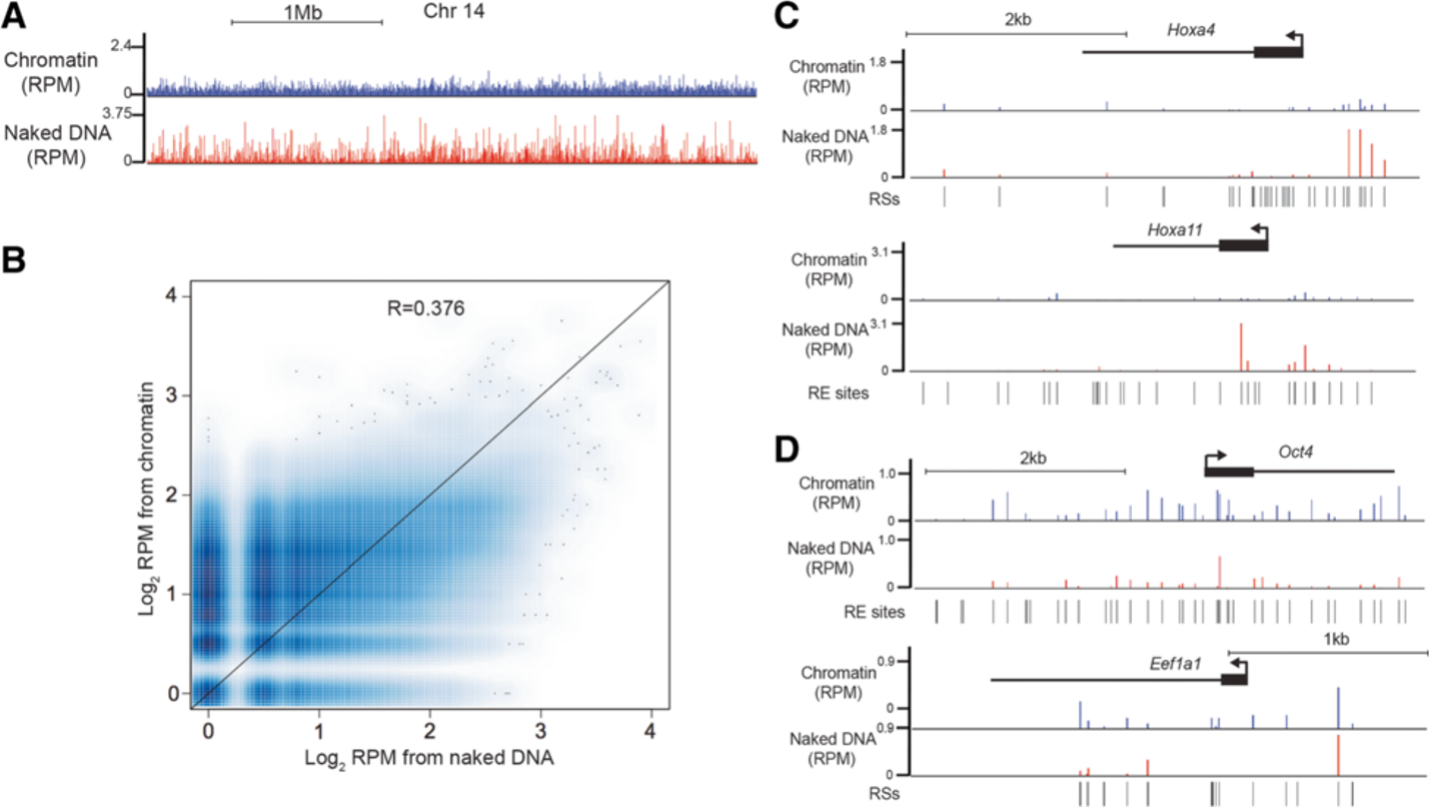
**Comparison of RED-seq to naked DNA digestion. (A)** RE accessibility reads from mouse ESC chromatin (top) and naked DNA (bottom) from a 3 Mb region of chromosome 14 (Chr14). Shown are normalized reads per million (RPM). **(B)** Scatterplot of RE accessibility [Log_2_(RPM)] for Chr14 from chromatin relative to naked DNA. **(C)** RE accessibility from chromatin and naked DNA of two *Hox* genes, *Hoxa4* and *Hoxa11*, which are silent in ESCs. Dotted lines highlight the genomic regions with RE accessibility differences apparent between chromatin and naked DNA. **(D)** RE accessibility from chromatin and naked DNA of two highly expressed genes in ESCs, *Oct4* and *Eef1a1*.

We developed a software package (also named REDseq; available as a Bioconductor package) to assign each read to a unique RS in the mouse genome (see Methods for details), and count the relative cut frequency per site corresponding to normalized read counts assigned to each RS. As we observed by electrophoresis of digested naked DNA or chromatin (Figure 1B), average RE accessibility, as measured by relative cut frequency per RS, was reduced in the chromatin library relative to naked DNA at most sites (Figure 2A). As expected, due to the fact that cutting frequency at each RS was normalized to total reads in each library, we observed fragments derived from some RSs that were more abundant in the chromatin library than the naked DNA library. In addition, cleavage within the naked DNA library was not uniform at all RSs (Figure 2A), likely due to the fact that fragments generated by two Sau96I cleavages within close proximity are selected against during library preparation, which eliminates small DNA fragments. This is less of a concern in chromatin samples, in which cleavage at most RSs is suppressed. Furthermore, we did not observe a strong correlation between the reads from chromatin DNA and naked DNA (R = 0.376), confirming that the degree of RE digestion at most sites was different between chromatin and naked DNA (Figure 2B). Thus, RED-seq accurately reflects inhibition of RE accessibility by the presence of chromatin *in vivo*.

### Active genes and nucleosome-free regions are highly accessible

RE accessibility in promoter-proximal regions is usually correlated with gene expression [36–38]. Homeobox (Hox) genes encode key developmental TFs that are not expressed in ESCs [39]. We observed low levels of RE accessibility around *Hox* genes relative to surrounding regions and normalized naked DNA reads (Figure 2C). In contrast, for genes that are highly expressed in ESCs (*Oct4*, *Eef1a1*), RE accessibility was elevated within upstream regulatory regions and surrounding transcriptional start sites (TSSs) (Figure 2D). Overall, these results showed that enhanced RE accessibility was generally associated with transcriptional activity, consistent with previous data.

**Figure 3:**
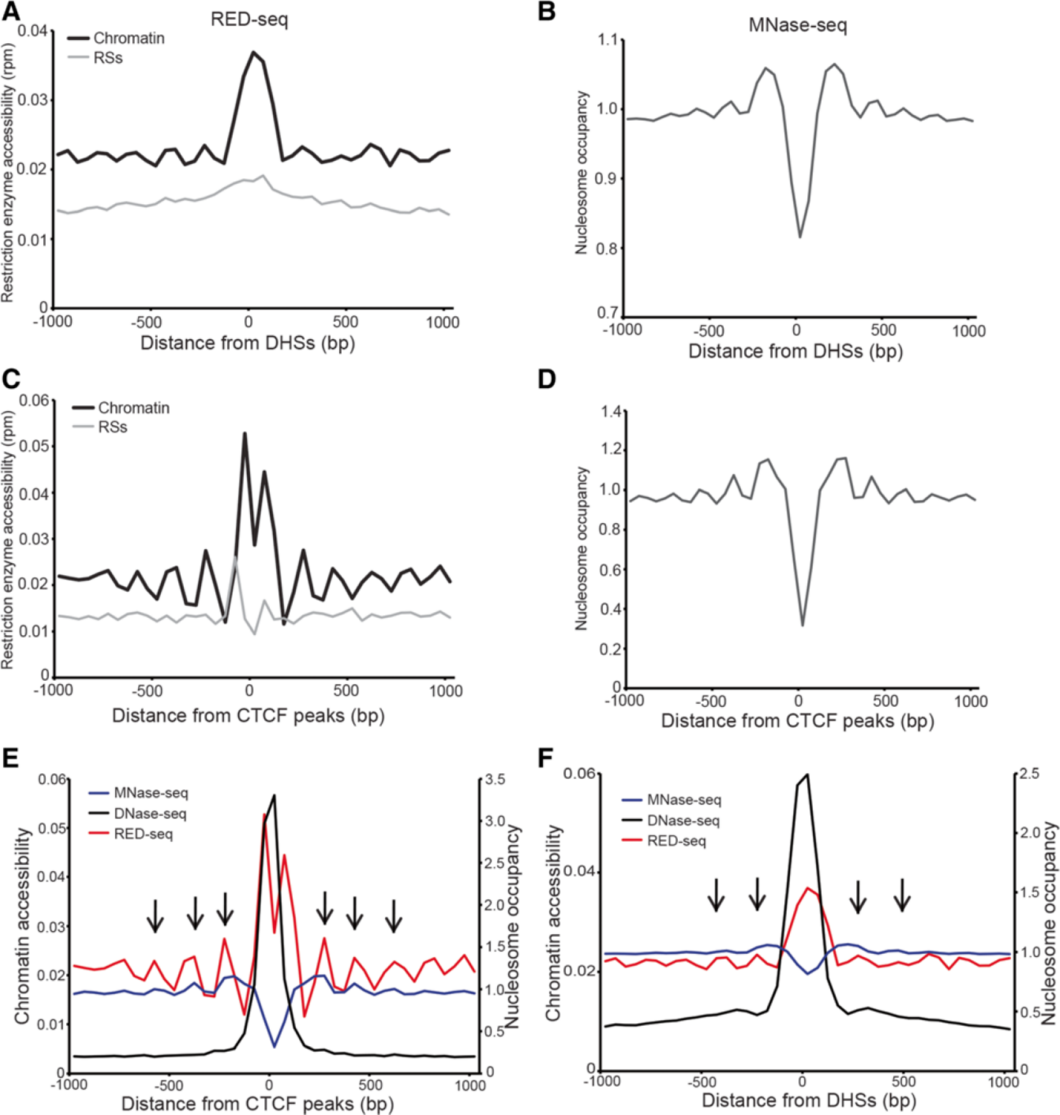
**RED-seq captures the enhanced accessibility of open chromatin regions.** Average RE accessibility **(A, C)** and nucleosome occupancy **(B, D)** [GEO:GSM1400766] of indicated chromatin domains. RED-seq or MNase-seq data are aligned on the centers of all peaks of DHSs **(A-B)**, or CTCF binding sites **(C-D)**, and averaged within a 2 kb region (-1000 to +1000 bp from the peaks). Normalized RE accessibility and RS density are shown. RE accessibility was normalized as in Figure 2. There are 159,331 DHSs [GEO:GSM1014154] **(A-B)**, and 15,657 CTCF binding sites [GEO:GSE11431] **(C-D)** plotted. **(E-F)** Chromatin accessibility determined by RED-seq or DNase-seq and nucleosome occupancy are shown surrounding CTCF binding sites **(E)** or DHSs **(F)**. Arrows indicate the phased peaks of RE accessibility found within linker regions.

DNase I is frequently used to identify open chromatin/nucleosome-free regions of the genome, and many gene regulatory elements are hypersensitive to DNase I [21, 22, 40, 41]. Therefore, we next examined the frequency of RED-seq reads surrounding annotated DHSs in ESCs. Since RSs are non-uniformly distributed throughout the genome, we compared RE accessibility averaged over all DHSs to average RS density to test whether DHSs were generally accessible or inaccessible. We found that RE accessibility over DHSs was strongly enhanced relative to the RS density surrounding these regions (Figure 3A). Similar results were observed in RED-seq maps of ESCs that combine Sau96I and a second RE, DdeI, validating these results (Additional file 1). Furthermore, our re-analysis of published NA-seq data from human NB-4 leukemia cells [42] revealed a similar pattern at DHSs, further confirming these results (Additional file 2). DHSs are typically nucleosome-depleted and highly transcribed, relative to DNase I-insensitive regions [21, 22, 40, 41]. Therefore, we compared our RED-seq data to nucleosome occupancy maps previously obtained by deep sequencing of nucleosome-sized DNA fragments protected from digestion by micrococcal nuclease (MNase-seq) [43], and found that nucleosomes were strongly depleted over DHSs (Figure 3B), consistent with the higher RE accessibility we observed.

Next, we compared RE accessibility surrounding the binding sites of two key TFs in ESCs. CTCF is a sequence-specific insulator binding protein with important roles in regulation of imprinted gene expression [44, 45] and higher-order chromatin structure [46]. RE accessibility was enriched within the regions surrounding CTCF (Figure 3C, Additional files 1 and 2). As previously reported [47, 48], CTCF binding sites are depleted of nucleosomes, with well-positioned nucleosomes flanking the nucleosome-free regions (Figure 3D), explaining the higher accessibility we observed at these sites. Interestingly, for highly abundant nucleosome-free regions such as CTCF binding sites and DHSs, RED-seq also revealed nucleosome phasing around nucleosome-depleted regions, with smaller phased peaks of RE accessibility found within linker regions (Figure 3E-F). Since the majority of inter-nucleosomal linkers are relatively small (averaging approximately 30 bp in ESCs [49], this phasing is not apparent using DNase-seq [29] which is specialized for identification of long stretches of nucleosome-free DNA (Figure 3E-F). Together these results show that while the resolution of RED-seq at the level of individual loci is variable and depends on the frequency of RSs at each locus, when averaged over thousands of loci RED-seq not only identifies large nucleosome-free regions identified by DNase-seq, but can also probe DNA linker regions within nucleosome arrays.

### Remodeling of chromatin accessibility during differentiation

**Figure 4:**
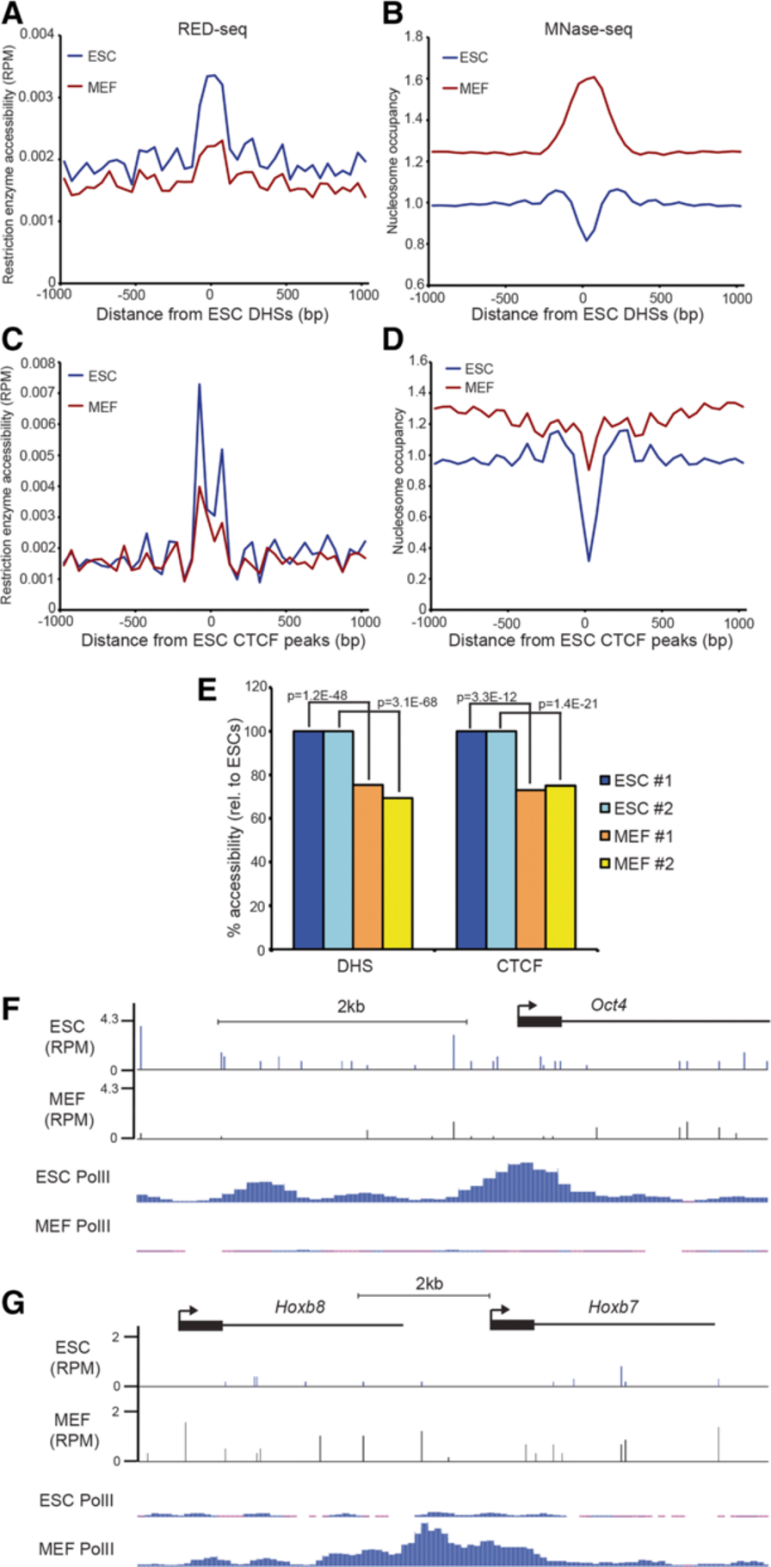
**Cell type-specific differences in chromatin accessibility. (A)** Average RE accessibility of ESCs (blue) or MEFs (red) shown relative to DNase I hypersensitive sites (DHSs) identified in ESCs [GEO:GSE46588]. **(B)** Nucleosome occupancy of the same regions is shown for ESCs [GEO:GSM1400766] and MEFs [GEO:GSM1004654]. **(C)** Average RE accessibility and **(D)** nucleosome occupancy surrounding CTCF binding regions in ESCs [GEO:GSE11431] are shown for ESCs and MEFs. **(E)** Average accessibilities over DHSs and CTCF binding sites were quantified for biological replicate experiments from –200 to +200 bp with respect to the indicated feature. P-values indicating statistical significance of accessibility between ESCs and MEFs are indicated. **(F, G)** RE accessibility of ESCs and MEFs surrounding the *Oct4* gene **(F)** and two genes within the *Hoxb* cluster **(G)**. RNA Polymerase II (RNA PolII) ChIP-seq reads [GEO:GSE29184] from ESCs and MEFs are shown for the same regions. RED-seq and MNase-seq data are plotted as in Figure 3.

ESC chromatin structure is relatively dynamic and is depleted of large blocks of heterochromatin, unlike many differentiated cell types, suggesting that major alterations in chromatin structure that accompany cellular differentiation may be important for lineage commitment [16]. To study chromatin accessibility during differentiation, we first tested whether RED-seq could identify distinct RE accessibility patterns in different cell types by comparing chromatin accessibility in ESCs and mouse embryonic fibroblasts (MEFs). We found that, in MEFs, nucleosome occupancy was increased and RE accessibility decreased at ESC-specific DHSs (Figure 4A-B), consistent with the widespread differences in chromatin structure and gene expression between these two cell types. As with DHSs, RE accessibility at sites of CTCF binding in ESCs was reduced in MEFs (Figure 4C-D), and these results were consistent in biological replicate RED-seq libraries from both cell types (Figure 4E). Finally, we examined RE accessibility within regions surrounding TSSs in both cell types. TSS-proximal regions of actively transcribed genes are usually nucleosome-depleted and the degree of nucleosome-depletion correlates with transcriptional activity at many genes. As expected, RE accessibility was higher in ESCs than in MEFs surrounding the TSSs of genes that were highly expressed in ESCs (Figure 4F), whereas genes highly expressed in MEFs were generally more accessible in MEFs (Figure 4G). These data confirmed that RED-seq could identify differences in chromatin accessibility between two distinct cell types that reflected differences in TF binding and gene expression.

**Figure 5:**
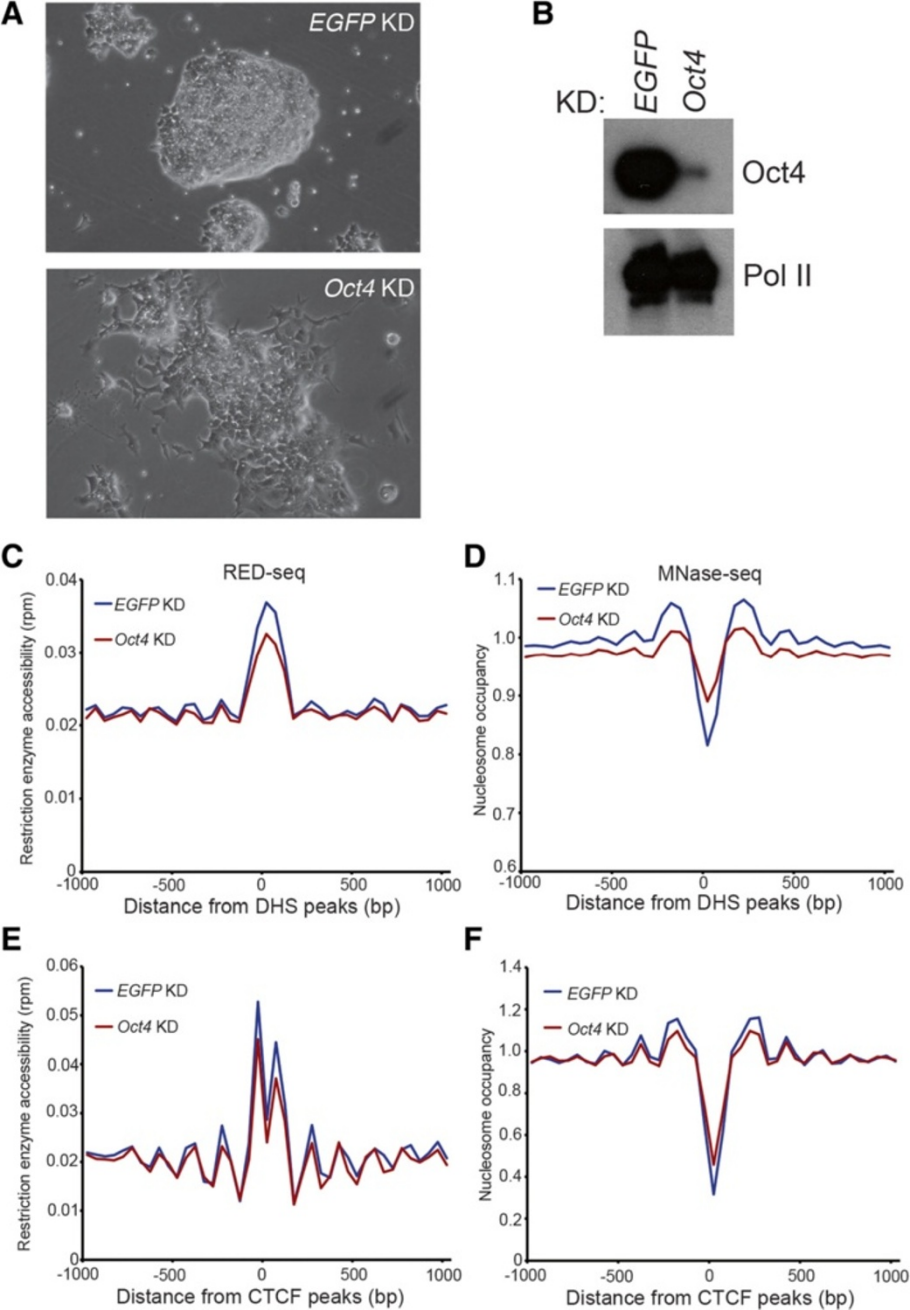
**Alterations in RE accessibility during ESC differentiation. (A)** Brightfield images of control (*EGFP*) or *Oct4* KD ESC colonies indicate colony flattening and elongated cellular morphology upon *Oct4* depletion. **(B)** Western blot of Oct4 in control (*EGFP*) or *Oct4* KDs, indicating KD efficiency. RNA Polymerase II blot (Pol II) is shown as a loading control. **(C, E)** Average RE accessibility upon *EGFP* or *Oct4* KD is shown relative to DHSs **(C)**, or CTCF binding sites **(E)**. **(D, F)** MNase-seq data. Nucleosome occupancy over DHSs **(D)**, or CTCF binding sites **(F)**. RED-seq and MNase-seq data are plotted as in Figure 3.

Next, to test whether we could observe more subtle changes in chromatin structure during cellular differentiation, we differentiated ESCs by RNAi-mediated knockdown (KD) of the ESC pluripotency TF Oct4. We chose this differentiation model since, unlike most other methods of differentiation that generate heterogeneous mixtures of many different cell types from all three germ layers, *Oct4* KD robustly induces trans-differentiation to trophectoderm specifically [50]. Consistent with previous reports [50], *Oct4* KD promoted ESC differentiation to cells with trophoblast morphology (Figure 5A-B). Using RED-seq, we found that RE accessibility was decreased upon *Oct4* KD near ESC DHSs and CTCF binding sites (Figure 5C, E). Although the reduction in DNA accessibility upon *Oct4* KD was not as severe as in MEFs, we also observed slightly increased nucleosome occupancy by MNase-seq upon *Oct4* KD at ESC DHSs and CTCF binding sites (Figure 5D, F), consistent with the decrease in RE accessibility that we observed in these regions.

**Figure 6:**
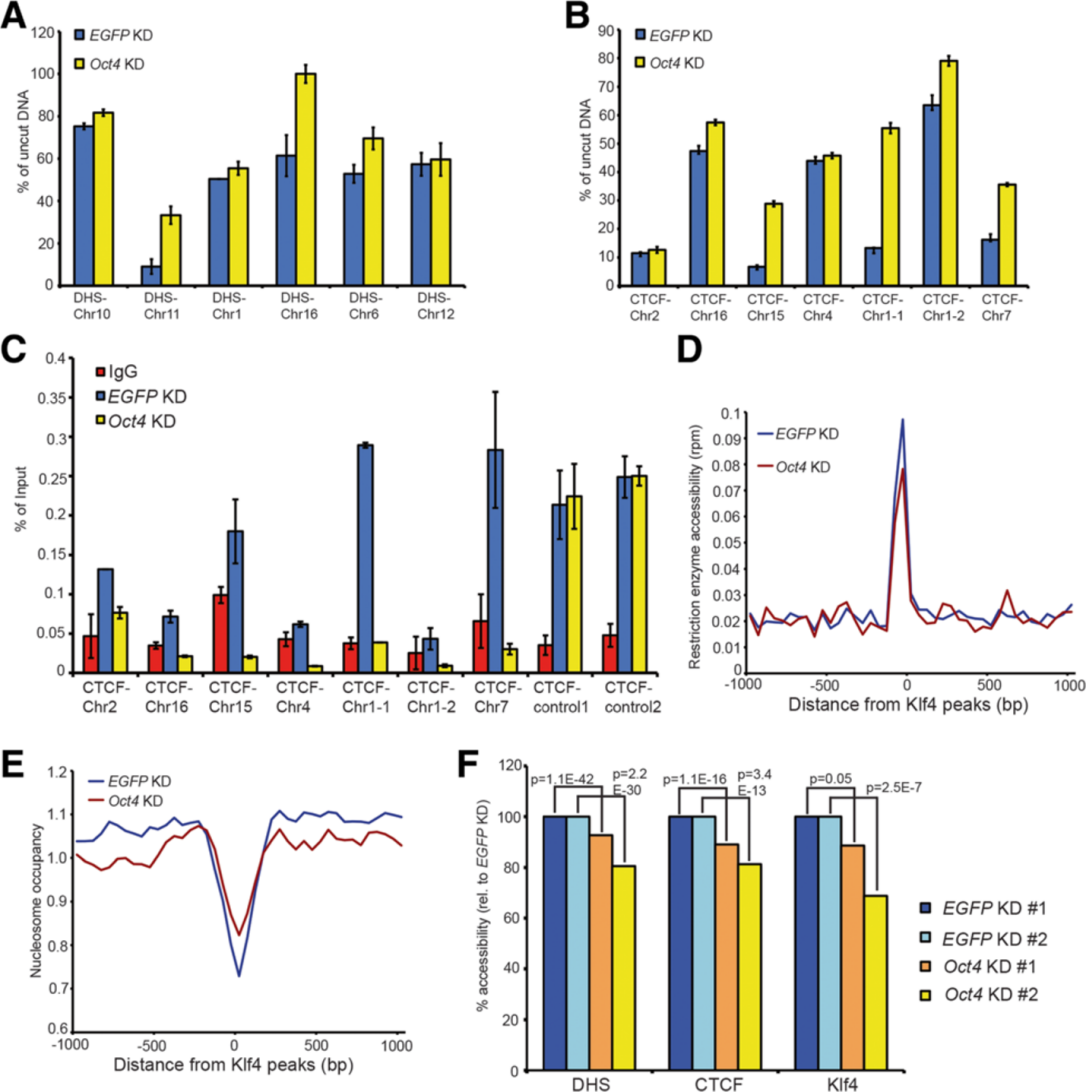
**Loss of chromatin accessibility at some CTCF binding sites correlates with reduced CTCF binding upon ESC differentiation. (A)** Differences in RE accessibility at specific DHSs were confirmed by qPCR across an RS of interest at each locus. Remaining uncut DNA after RE digestion of each indicated KD is shown for several DHSs that exhibited accessibility differences by RED-seq. Data are normalized to uncut genomic DNA. **(B)** Confirmation of restriction enzyme accessibility surrounding CTCF binding sites, as in **(A)**. **(C)** CTCF ChIP-qPCR data are shown for the indicated KDs at several CTCF binding sites. Controls are CTCF binding sites in which accessibility did not change upon *Oct4* KD. Data are presented as a percentage of input DNA. Shown are the mean ± SD of three technical replicates from one representative experiment of two biological replicates performed. **(D-E)** RED-seq data **(D)** and MNase-seq data **(E)** over Klf4 binding sites, plotted as in Figure 3. **(F)** Average accessibilities over DHSs, CTCF binding sites, and Klf4 binding sites were quantified for biological replicate KD experiments from –200 to +200 bp with respect to the indicated feature. P-values indicating statistical significance of accessibility between *EGFP* KD and *Oct4* KD are indicated.

To validate these results, we used quantitative PCR (qPCR) to determine the fraction of uncut (protected) DNA after RE digestion, probing several ESC DHSs and CTCF binding sites. Consistent with the RED-seq results, higher levels of uncut DNA were observed upon *Oct4* KD at most sites tested (Figure 6A-B). Furthermore, we tested CTCF binding at the same regions by ChIP-qPCR, and observed a reduction in binding upon *Oct4* KD wherever chromatin accessibility decreased, whereas control CTCF binding sites that showed no difference in accessibility upon *Oct4* KD showed no decrease in CTCF binding (Figure 6C). These data indicate that CTCF binding and RE accessibility are inter-dependent. Next, we observed that RE accessibility surrounding the binding sites of the ESC TF Klf4 was also reduced upon *Oct4* KD (Figure 6D), with concomitant increases in nucleosome occupancy over these sites (Figure 6E). Finally, we found the alterations in accessibility we observed over DHSs, CTCF binding sites, and Klf4 binding sites were consistent in two biological RED-seq replicates from each KD (Figure 6F), further validating these results. These results suggest that, during differentiation, many enhancers that are protected from nucleosome deposition in ESCs (presumably by TF binding) become occupied by nucleosomes, leading to decreased RE accessibility. Taken together, RED-seq not only detects large differences in chromatin accessibility between distinct cell types (ESCs vs MEFs) but also tracks more subtle changes that occur during differentiation (control vs *Oct4* KD ESCs).

### Altered accessibility of nucleosomes harboring distinct histone variants

Genomic regions that are dynamic (i.e. experience relatively rapid exchange of chromatin proteins) are frequently marked with specific histone modifications and/or histone variants [51]. However, using traditional methods such as DNase-seq or FAIRE-seq, it is difficult to identify differences in chromatin accessibility that correlate with the presence of dynamic nucleosomes, because these regions are not nucleosome-free. In principle, RED-seq does not share these limitations, due to the fact that a single RE cleavage is all that is necessary for inclusion in a RED-seq library (Figure 1A). Therefore, we examined the accessibility of regions enriched for dynamic histone variants/modifications using RED-seq.

**Figure 7:**
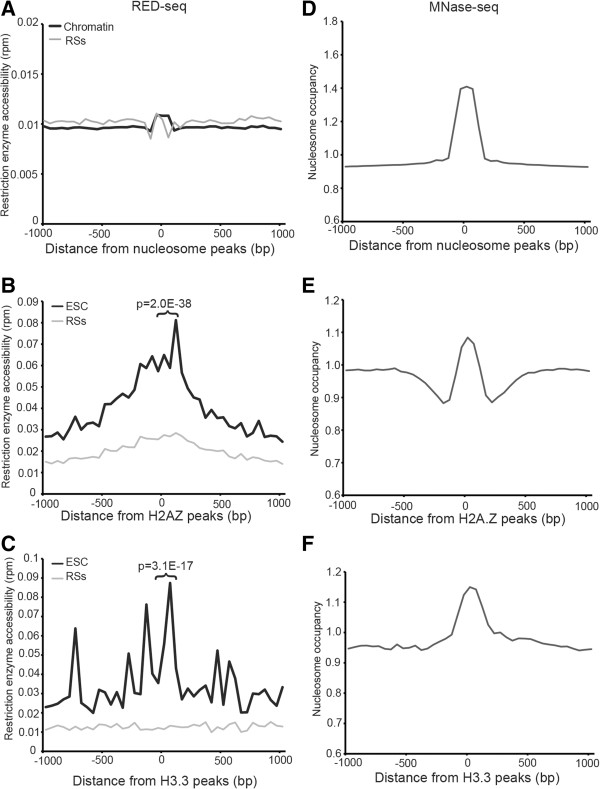
**Enhanced accessibility of DNA bound by H2A.Z-containing nucleosomes.** Average RE accessibility **(A-C)** and nucleosome occupancy **(D-F)** shown relative to 320,135 randomly selected nucleosomes **(A, D)**, 39,437 H2A.Z-containing nucleosomes [GEO:GSE34483] **(B, E)**, or 8,287 H3.3-containing nucleosomes [GEO:GSE16893] **(C, F)**. Data are plotted as in Figure 3. P-values indicating statistical significance of accessibility between H2A.Z and average nucleosome profiles, as well as H3.3 and average nucleosomes are indicated.

To establish a baseline for the examination of different types of nucleosomes, we first determined the average accessibility of a random distribution of nucleosomes across the genome. To this end, we randomly selected 1% of all nucleosomal footprints from an MNase-seq library prepared from ESCs, and plotted the average RED-seq and MNase-seq profiles within a 2 kb window surrounding their positions. Consistent with the fact that nucleosome-bound DNA is relatively inaccessible to nuclear factors, we observed a low level of RE accessibility surrounding the peak of bulk nucleosomes, relative to RS density (Figure 7A). Therefore, as expected, nucleosome-free DNA, like that underlying DHSs and TF binding sites, is generally more accessible than nucleosomal DNA.

Next, we tested whether the accessibility of nucleosome variants that harbor particular histone modifications or histone variants were identical to that of bulk nucleosomes. The two nucleosomes surrounding TSSs (referred to as +1 and -1 nucleosomes) are frequently marked by histone variants H2A.Z and H3.3 [6, 12–14]. Nucleosomes harboring these variants have been found to be extractible from chromatin at lower salt than is required for canonical nucleosomes [6, 12], raising the possibility that they may be more highly accessible in general. H2A.Z is enriched surrounding the TSSs of many eukaryotic genes, and also found within active enhancers in mammalian cells [52]. Furthermore, H2A.Z-marked nucleosomes protect smaller footprints of DNA than canonical nucleosomes, in support of the hypothesis that that these nucleosomes are more intrinsically accessible [14]. In ESCs, H2A.Z is found near approximately 84% of all TSSs, including those of many silent genes [53]. Interestingly, we observed increased RE accessibility over the center of the H2A.Z peaks relative to both RS density and surrounding regions ± 1 kb from the peaks of H2A.Z enrichment (Figure 7B), suggesting that H2A.Z-containing nucleosomes are generally more accessible than canonical nucleosomes. Next, we examined H3.3, which is enriched near the TSSs of both active and silent genes, as well as within gene bodies of highly expressed genes, and is incorporated into chromatin in a replication-independent manner [54–56]. Like H2A.Z, we found that RE accessibility over H3.3 peaks was elevated relative to RS density (Figure 7C). These data suggest that DNA wrapped around H2A.Z- and H3.3-marked nucleosomes is more accessible than DNA found within the majority of nucleosomes genome-wide that lack these histone variants.

We considered the possibility that the elevated RE accessibilities observed over peaks of H2A.Z enrichment and broad regions surrounding H3.3 were due to reduced nucleosome occupancy at these sites. However, while the average occupancies of H2A.Z- and H3.3-containing nucleosomes were slightly lower than bulk nucleosomes (compare the peak heights in Figure 7D-F), these modest differences are insufficient to account for the greater than 5-fold increase in accessibility observed over H2A.Z and H3.3 peaks observed by RED-seq.

**Figure 8:**
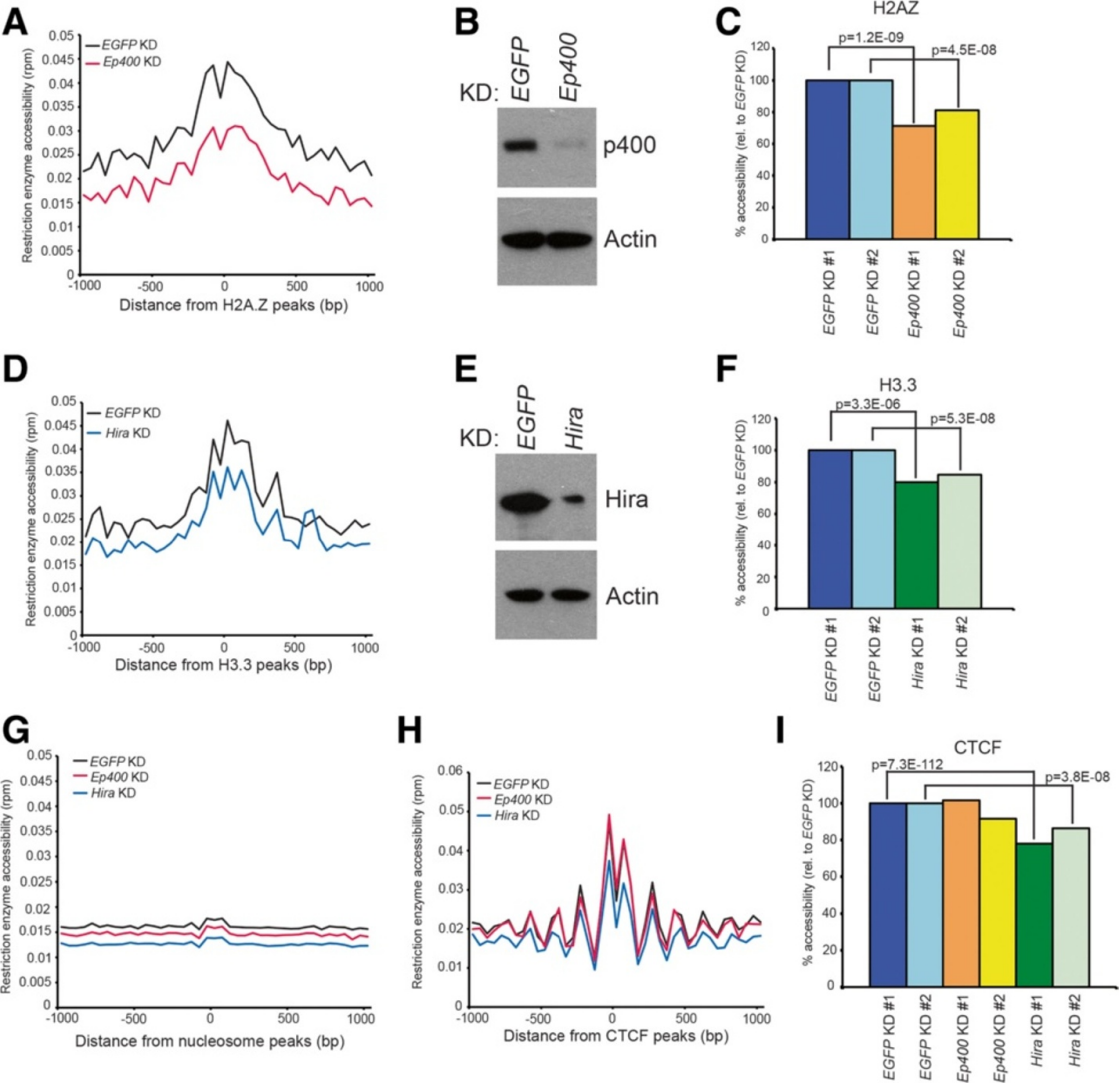
**Factors required for H2A.Z or H3.3 deposition are required for enhanced accessibility of regions normally bound by these histone variants. (A)** Chromatin accessibility determined by RED-seq averaged over regions of the genome bound by H2A.Z, as in Figure 7. Shown are control (*EGFP* KD) and *Ep400* KD ESCs. **(B)** Western blot of p400 in control (*EGFP*) or *Ep400* KDs, indicating KD efficiency. Actin is shown as a loading control. **(C)** Average accessibilities over H2A.Z-marked nucleosomes were quantified for biological replicate experiments from –200 to +200 bp with respect to the H2A.Z peak. P-values indicating statistical significance of accessibility between *EGFP* and *Ep400* KDs are indicated. **(D)** Chromatin accessibility determined by RED-seq averaged over regions of the genome bound by H3.3. Shown are control (*EGFP* KD) and *Hira* KD ESCs. **(E)** Western blot of Hira in control (*EGFP*) or *Hira* KDs, indicating KD efficiency. Actin is shown as a loading control. **(F)** Average accessibilities over H3.3-marked nucleosomes were quantified for biological replicate experiments from –200 to +200 bp with respect to the H3.3 peak. P-values indicating statistical significance of accessibility between *EGFP* and *Hira* KDs are indicated. **(G)** Effects of *Ep400* or *Hira* KD on average nucleosome accessibility shown by plotting RED-seq data over the same 320,135 randomly selected nucleosomes as in Figure 7A. **(H)** Effects of *Ep400* or *Hira* KD on chromatin accessibility over CTCF binding sites, as in Figure 3C. **(I)** Average accessibilities over CTCF-binding sites were quantified for biological replicate experiments from –200 to +200 bp with respect to the peak of CTCF-binding. P-values indicating statistical significance of accessibility between *EGFP* and *Hira* KDs are indicated.

To validate these data, we examined chromatin accessibility upon KD of factors necessary for incorporation of H2A.Z or H3.3 into chromatin. In mammals, H2A.Z is incorporated into chromatin in part by p400 (gene name: *Ep400*), a homolog of the yeast Swr1 ATPase, whereas H3.3 incorporation depends in part on the HIRA (*Hira*) histone chaperone [57, 58]. We tested whether the enhanced chromatin accessibility observed at sites of H2A.Z and H3.3 deposition was reduced upon depletion of their respective loading factor, and found that the elevated accessibility we observed within regions of H2A.Z and H3.3 enrichment was partially lost upon *Ep400* KD or *Hira* KD, respectively (Figure 8A-F). When we examined alterations in chromatin accessibility upon *Ep400* or *Hira* KD over a random sampling of nucleosomes (as in Figure 7A), we observed only a modest decrease in accessibility, suggesting that the effects of *Ep400* or *Hira* KD are specific for nucleosomes containing H2A.Z or H3.3 (Figure 8G). Finally, we examined changes in chromatin accessibility due to *Ep400* or *Hira* KD over CTCF binding sites, due to the reported enrichment of H2A.Z- and H3.3-containing nucleosomes surrounding CTCF [13]. Interestingly, while *Hira* KD resulted in significantly reduced accessibility over CTCF binding sites, *Ep400* KD did not (Figure 8H), suggesting that either H3.3 plays a more important role than H2A.Z in regulation of chromatin structure near CTCF binding sites or that H2A.Z is incorporated into chromatin at these sites independently of p400. We observed consistent differences in accessibility over H2A.Z, H3.3, and CTCF binding sites in biological replicate KDs of *Ep400*, *Hira*, and *Hira*, respectively (Figure 8C, F and I), validating these data. Together, these results suggest that H2A.Z- and H3.3-containing nucleosomes are either more dynamic or more intrinsically accessible than canonical nucleosomes, consistent with their association with gene regulatory sequences.

## Discussion

Utilizing an adaptation of a decades-old, quantitative technique for probing chromatin accessibility, we probed the chromatin structure of ESCs and differentiated cells, observing differences in chromatin accessibility in distinct regions of the genome, as well as in different cellular states. We found that both the level of nucleosome occupancy and the presence of specific histone variants at individual loci affected the level of chromatin accessibility we observed at each site.

Over the past several years, DNase-seq and FAIRE-seq have been used to identify regions of open chromatin structure within cells. One limitation of these methods is that only nucleosome-depleted regions of DNA are typically identified. Interestingly, while RED-seq identified nucleosome-depleted regions as well, we also observed differences in chromatin accessibility within nucleosomes that harbor specific histone variants, detecting increased RE accessibility in genomic regions enriched for histones H2A.Z and H3.3. Therefore, unlike previous methods, RED-seq not only measures general chromatin “openness” but also identifies highly dynamic regions of the genome, even if they are not nucleosome-free. We believe that this feature – the ability to quantify accessibility of DNA within nucleosome-bound regions – best distinguishes RED-seq from complementary approaches such as MNase-seq and DNase-seq, which do not probe intranucleosomal accessibility.

The increased accessibility of DNA within H2A.Z- and H3.3-containing nucleosomes is due to the histone variants themselves rather than some unrelated feature of chromatin structure within these regions of the genome, since depletion of H2A.Z and H3.3 loading factors strongly reduced the accessibility of the underlying DNA. Although H2A.Z and H3.3 are also enriched near TSSs, these histone variants are also found within multiple other genomic domains. Indeed, we find that accessibility over CTCF binding sites was reduced upon KD of the H3.3 deposition factor, *Hira*, suggesting that H3.3 incorporation within nucleosomes surrounding CTCF binding sites may be important for CTCF binding and/or function.

Chromatin structure is dramatically altered during cellular differentiation. By examining regions of the genome enriched for histone modifications, TFs, or chromatin regulators, RED-seq could identify differences in chromatin structure within functionally distinct regions of the genome during ESC differentiation. We found that RE accessibility decreased at many CTCF binding sites upon *Oct4* KD and that this decrease correlated with a decrease in CTCF occupancy and an increase in nucleosome occupancy. These differences were even more apparent when comparing ESCs with MEFs. Together, these results suggest that loss of TF binding during differentiation is coincident with deposition of nucleosomes at these sites, leading to loss of chromatin accessibility.

Besides chromatin structure, restriction enzymes have been widely used in biological assays for single nucleotide polymorphisms (SNPs) [59, 60] and DNA methylation [61] at individual loci, by virtue of their inhibitory effect on RE cleavage. Therefore, a genome-wide method to directly quantify differences in RE cleavage would be highly desirable in these assays. Our method of directly purifying RE-digested sequences and quantifying RE cleavage at each site by high-throughput DNA sequencing could be easily adapted to perform these types of studies. Thus, we believe that RED-seq will be a valuable tool for not only the measurement of chromatin accessibility and dynamics, but also the study of any other phenomena that alter RS cleavage by REs.

## Conclusions

We developed RED-seq, an unbiased probe of chromatin accessibility, and utilized this technique to probe chromatin structure genome-wide in mouse ESCs and differentiated cells. Unlike more widely used methods that positively identify broad domains of open chromatin structure, RED-seq not only identifies open chromatin domains, but also uncovers differences in DNA accessibility within the vast majority of the genome that is not found within a large nucleosome-free region. By examining the accessibility of DNA wrapped within distinct nucleosome variants, we found that H2A.Z- and H3.3-containing nucleosomes were more accessible than the genomic average, providing *in vivo* evidence that these nucleosomes may be more dynamic than canonical nucleosomes. Therefore, RED-seq provides unique insights into chromatin structure that are missed by more widely utilized approaches.

## Methods

### Cells

The murine ESC line used in this study was E14 [62]. Mouse embryonic fibroblasts (MEFs) used in this study were immortalized by serial passaging, following a 3 T3 protocol, to minimize day-to-day differences in these cells due to their passage number. Mice used in derivation of MEFs were housed in a specific pathogen-free facility at the University of Massachusetts Medical School, and all experiments were performed in strict accordance with the recommendations of the Institutional Animal Care and Use Committee at the University of Massachusetts Medical School (approval #2165-13).

### Preparation of RED-seq libraries

One million cells were used to construct RED-seq libraries. Cells were washed, pelleted, and resuspended in swelling buffer (10 mM Tris pH8.0, 85 mM KCl, 0.5% NP-40, 10 mM MgCl_2_) with 100 units of Sau96I (NEB) and incubated in a thermomixer (Eppendroff) at 37°C for 1 hour, shaking at 900 rpm. (For testing whether two REs might increase coverage, in one experiment 100 units of Sau96I and 50 units of DdeI were used in digestion). Digestion was terminated by adding 40 μl of 10% SDS and 20 μl of 0.5 M EDTA and the chromatin was treated with proteinase K (Ambion) overnight at 55°C. Digested DNA was purified using phenol/chloroform/isoamyl alcohol extractions and precipitated at -80°C for 1 hour. Digested DNA samples were end-repaired and A-tailed as described [63], and ligated with biotinylated and barcoded adaptors. DNA was purified using Zymo Research DNA clean and concentrate columns following each enzyme reaction. The biotin-adaptor ligated DNA was sonicated in a Covaris sonicator (S220) to generate DNA peak fragments of 200 bp, on average. The sonicated DNA samples were then end-repaired, A-tailed, and ligated with non-biotinylated adaptors. The ligation samples were loaded on 2% agarose gel and DNA was purified within a size range of roughly 200-350 bp in length. Gel-purified DNA was diluted to 250 μl with streptavidin binding buffer (20 mM HEPES pH 7.6, 500 mM NaCl, 1 mM EDTA, 0.02% NP-40) and incubated with 30 μl of pre-washed streptavidin magnetic beads (NEB) at room temperature for 1 hour. After magnetic separation, the supernatants were removed, and the beads were washed additional three times with streptavidin binding buffer. DNA was eluted from streptavidin magnetic beads by adding 20 μl of 0.1X TE and heating at 60°C for 3 minutes. The elution was repeated three times. The adaptor-ligated material was then PCR amplified with Phusion polymerase (NEB) using 16 cycles of PCR and its concentration was determined using a NanoDrop (Thermo). The integrity of each library was confirmed by sequencing 10-20 individual fragments per library. Libraries with different barcodes were pooled together and single-end sequencing (50 bp) was performed on an Illumina HiSeq2000 at the UMass Medical School deep sequencing core facility.

For most RED-seq libraries (*GFP*, *Oct4*, *Ep400* and *Hira* KD), we added one further modification in which the sequence of the biotinylated adapters and the second, non-biotinylated, adapters were modified such that after PCR amplification of the libraries, only the end that was ligated to the biotinylated adapter would be sequenced in a single-end sequencing run (Additional file 3). Although this alteration makes the data analysis slightly simpler, the two methods provide essentially identical results.

### Preparation of MNase-seq libraries

MNase-library preparation was adapted from Henikoff et al. [27]. Formaldehyde cross-linked cells were pelleted and washed twice with PBS. Cell pellets were resuspended in MNase lysis buffer (10 mM Tris pH 7.5, 10 mM NaCl, 3 mM MgCl_2_, 0.5% NP-40, 1 mM CaCl_2_, and protease inhibitors) and treated with 10 units/10^6^ cells of microccocal nuclease (Roche) for 5 minutes at 37°C. The reaction was stopped with 10 mM EDTA. Nuclei were then incubated with RNaseA (Ambion) for 4 hours at 4°C with rotation followed by incubation with proteinase K (Ambion) overnight at 55°C. DNA was then isolated by Phenol: Chloroform:Isoamyl Alcohol (PCI) and EtOH precipitation. Equal MNase digestion was confirmed by examining DNA size fragments through electrophoresis on a 2% agarose gel and through bioanalyzer analysis. After phosphatase (NEB) treatment, digested DNA was end-repaired and A-tailed, with PCI extraction and EtOH precipitation following each enzyme reaction. Adaptors were ligated and DNA was size selected using Agencourt Ampure XP beads (Beckman Coulter), as previously described [27]. Equal library sizes were confirmed through electrophoresis on a 2% agarose gel and through bioanalyzer analysis. Sequencing of 10 fragments per library confirmed the integrity and libraries were sent for paired-end (100 bp) high throughput sequencing using an Illumina HiSeq at the UMass Medical School sequencing facility. Reads were mapped to the mouse genome (mm9) using Bowtie2 and uniquely mapped reads were used for further analysis.

### Data analysis

#### Assignment of reads to individual RSs

Sequence reads were binned according to the 4 bp barcode present at the beginning of each sequence using a custom Perl script. Sequences with barcodes removed were mapped to the mouse genome (mm9) using Bowtie-0.12.7 [64] with parameters set as -n 2 -l 28 -M 1 --best --strata (i.e. uniquely mapped with at most 2 mismatches at the left 28 bp seed region). Assignment of aligned sequences to individual restriction enzyme cut sites (REs) and differential cut analysis were performed using the Bioconductor package REDseq, developed by us. The ChIPpeakAnno package [65] was used to annotate the differentially cut sites to the nearest genes. Surprisingly, we found that the GGTCC sequence was cleaved more efficiently by Sau96I than GGACC, GGGCC, or GGCCC in digestions of chromatin or naked DNA control samples. This altered specificity may be due to the different buffer conditions used for digestion of chromatin (which are optimized for permeabilization of cells) relative to the optimal buffer conditions for Sau96I digestion recommended by the manufacturer. However, this phenomenon was observed in all samples, independent of cell type or KD, and therefore does not affect any comparisons of accessibility.

#### Aggregation of RED-seq data at specific genomic regions

Data for DNase I hypersensitive sites was downloaded from mouse ENCODE Project (UCSC). ChIP-seq data for H2A.Z (GSE34483), H3.3 (GSE16893), H3K4me3 (GSE12241) were downloaded from GEO datasets (NCBI) and analyzed in HOMER software suite [66]. The MNase-seq data in ESCs was obtained from Carone et al. [43]. The enrichment regions were identified by using the “findPeaks” command in HOMER with default setting (1. fold enrichment over local tag count, default: 4.0. 2. Poisson p-value threshold relative to local tag count, default: 0.0001 3. False discovery rate, default = 0.001). For the binding sites of different TFs (CTCF and Klf4) in ESCs, the enriched regions were obtained from GEO datasets (GSE11431) and converted to mm9 by LiftOver (UCSC Genome Bioinformatics Group).

#### Calculation of restriction enzyme accessibility

RED-seq data was processed in HOMER by using “annotatePeaks” command to bin the regions of interest in 50 bp windows and sum the reads within each window. Average RE accessibility was calculated by normalizing the reads in each window to total reads, dividing by the number of regions of interest, and presented in reads per million. To calculate the genome-wide distribution of restriction enzyme sites, we manually assigned one read to each site and calculated average RE accessibility as mentioned above.

### Measurement of restriction enzyme accessibility at individual loci

DNA from RE-digested chromatin was prepared as above, up to the first DNA purification step (prior to library preparation). DNA was resuspended in 50 μl of 0.1X TE and 10 ng of DNA subjected was to quantitative PCR (qPCR) using SYBR FAST universal reagents (KAPA Biosystems) with specific primers (Additional files 4 and 5) flanking RSs of interest.

### RNAi

RNAi-mediated KD of *Oct4*, *p400*, *Hira* or *GFP* (control) was performed using esiRNAs as described [67, 68]. For differentiation experiments, *GFP* (control) or *Oct4* esiRNAs were transfected into ESCs using Lipofectamine 2000 (Invitrogen). Chromatin was isolated and used for RED-seq or MNase-seq library construction 5 days after transfection.

### Chromatin immunoprecipitation

ChIP samples were prepared as described [69]. Briefly, chromatin from *GFP* or *Oct4* KD ESCs was crosslinked, lysed and sonicated to generate 300-1000 base-pair fragments. 50 μl of Protein A Magnetic beads (NEB) were washed twice with PBS containing 5 mg/ml BSA and 10 μl of anti-CTCF antibody (Millipore) was coupled in 500 μl PBS with 5 mg/ml BSA overnight at 4°C. Immunoprecipitation was performed with antibody-coupled beads and sonicated supernatants in ChIP buffer (20 mM Tris-HCl pH8.0, 150 mM NaCl, 2 mM EDTA, 1% Triton X-100) overnight at 4°C. Magnetic beads were washed twice with ChIP buffer, once with ChIP buffer including 500 mM NaCl, 4 times with RIPA buffer (10 mM Tris-HCl pH 8.0, 0.25 M LiCl, 1 mM EDTA, 0.5% NP-40, 0.5% Na⋅Deoxycholate), and once with TE buffer (pH 8.0). Chromatin was eluted twice from washed beads by adding elution buffer (20 mM Tris-HCl pH 8.0, 100 mM NaCl, 20 mM EDTA, 1% SDS) and incubating for 15 minutes at 65°C. Crosslinking was reversed at 65°C for 6 hr and RNase A/T1 (Ambion) was added for 1 hr at 37°C followed by proteinase K (Ambion) treatment overnight at 50°C. ChIP-enriched DNA was purified using Phenol/Chloroform/Isoamyl alcohol extractions in phase-lock tubes. Then, chromatin was analyzed by qPCR as described above, using primers specific for CTCF sites of interest (Additional file 5).

### Data access

The genome-wide data sets generated in this study can be obtained from GEO [GEO:GSE51821].

## Electronic supplementary material

Additional file 1: **Testing RED-seq using two REs.** Average RE accessibility within a 2 kb region (-1000 to +1000 bp from the peaks) of DHSs or CTCF binding sites as measured by RED-seq using Sau96I and DdeI, plotted as in Figure 3. (TIFF 221 KB)

Additional file 2: **Re-analysis of NA-seq data.** Average RE accessibility within a 2 kb region (-1000 to +1000 bp from the peaks) of DHSs or CTCF binding sites from published NA-seq data [GEO:GSE30254], plotted as in Figure 3. (TIFF 205 KB)

Additional file 3: **Sequences of barcoded and biotinylated adaptors.** (PDF 42 KB)

Additional file 4: **Sequences of qPCR primers for DHSs.** (PDF 40 KB)

Additional file 5: **Sequences of qPCR primers for CTCF binding sites.** (PDF 48 KB)

## Abbreviations

RED-seq: Deep sequencing analysis of restriction enzyme digestion
MNase-seq: Deep sequencing of micrococcal nuclease footprints
DHS: DNase I hypersensitive site
DNase-seq: Deep sequencing of DHSs
FAIRE-seq: Deep sequencing of DNA fragments from FAIRE – Formaldehyde-Assisted Isolation of Regulatory Elements
TF: Transcription factor
ESC: Embryonic stem cell
MEF: Mouse embryonic fibroblast
RE: Restriction enzyme
RS: Restriction site
TSS: Transcription start site
H3K4me3: Histone H3 trimethylated on lysine 4.

## References

[1] Luger K, Richmond TJ (1998). DNA binding within the nucleosome core. Curr Opin Struct Biol.

[2] Abbott DW, Ivanova VS, Wang X, Bonner WM, Ausió J (2001). Characterization of the stability and folding of H2A.Z chromatin particles: implications for transcriptional activation. J Biol Chem.

[3] Bao Y, Konesky K, Park Y-J, Rosu S, Dyer PN, Rangasamy D, Tremethick DJ, Laybourn PJ, Luger K (2004). Nucleosomes containing the histone variant H2A.Bbd organize only 118 base pairs of DNA. EMBO J.

[4] Doyen C-M, Montel F, Gautier T, Menoni H, Claudet C, Delacour-Larose M, Angelov D, Hamiche A, Bednar J, Faivre-Moskalenko C, Bouvet P, Dimitrov S (2006). Dissection of the unusual structural and functional properties of the variant H2A.Bbd nucleosome. EMBO J.

[5] Thambirajah AA, Dryhurst D, Ishibashi T, Li A, Maffey AH, Ausió J (2006). H2A.Z stabilizes chromatin in a way that is dependent on core histone acetylation. J Biol Chem.

[6] Jin C, Felsenfeld G (2007). Nucleosome stability mediated by histone variants H3.3 and H2A.Z. Genes Dev.

[7] Luger K, Dechassa ML, Tremethick DJ (2012). New insights into nucleosome and chromatin structure: an ordered state or a disordered affair?. Nat Rev Mol Cell Biol.

[8] Watanabe S, Radman-Livaja M, Rando OJ, Peterson CL (2013). A histone acetylation switch regulates H2A.Z deposition by the SWR-C remodeling enzyme. Science.

[9] Li W, Nagaraja S, Delcuve GP, Hendzel MJ, Davie JR (1993). Effects of histone acetylation, ubiquitination and variants on nucleosome stability. Biochem J.

[10] Wang X, Hayes JJ (2008). Acetylation mimics within individual core histone tail domains indicate distinct roles in regulating the stability of higher-order chromatin structure. Mol Cell Biol.

[11] Chandrasekharan MB, Huang F, Sun Z-W (2010). Histone H2B ubiquitination and beyond: Regulation of nucleosome stability, chromatin dynamics and the trans-histone H3 methylation. Epigenetics.

[12] Henikoff S, Henikoff JG, Sakai A, Loeb GB, Ahmad K (2009). Genome-wide profiling of salt fractions maps physical properties of chromatin. Genome Res.

[13] Jin C, Zang C, Wei G, Cui K, Peng W, Zhao K, Felsenfeld G (2009). H3.3/H2A.Z double variant-containing nucleosomes mark “nucleosome-free regions” of active promoters and other regulatory regions. Nat Genet.

[14] Tolstorukov MY, Kharchenko PV, Goldman JA, Kingston RE, Park PJ (2009). Comparative analysis of H2A.Z nucleosome organization in the human and yeast genomes. Genome Res.

[15] Weintraub H, Groudine M (1976). Chromosomal subunits in active genes have an altered conformation. Science.

[16] Meshorer E, Misteli T (2006). Chromatin in pluripotent embryonic stem cells and differentiation. Nat Rev Mol Cell Biol.

[17] Meshorer E (2007). Chromatin in embryonic stem cell neuronal differentiation. Histol Histopathol.

[18] Crawford GE, Davis S, Scacheri PC, Renaud G, Halawi MJ, Erdos MR, Green R, Meltzer PS, Wolfsberg TG, Collins FS (2006). DNase-chip: a high-resolution method to identify DNase I hypersensitive sites using tiled microarrays. Nat Methods.

[19] Sabo PJ, Kuehn MS, Thurman R, Johnson BE, Johnson EM, Cao H, Yu M, Rosenzweig E, Goldy J, Haydock A, Weaver M, Shafer A, Lee K, Neri F, Humbert R, Singer MA, Richmond TA, Dorschner MO, McArthur M, Hawrylycz M, Green RD, Navas PA, Noble WS, Stamatoyannopoulos JA (2006). Genome-scale mapping of DNase I sensitivity in vivo using tiling DNA microarrays. Nat Methods.

[20] Wu C (1980). The 5’ ends of Drosophila heat shock genes in chromatin are hypersensitive to DNase I. Nature.

[21] Saragosti S, Moyne G, Yaniv M (1980). Absence of nucleosomes in a fraction of SV40 chromatin between the origin of replication and the region coding for the late leader RNA. Cell.

[22] Schones DE, Cui K, Cuddapah S, Roh T-Y, Barski A, Wang Z, Wei G, Zhao K (2008). Dynamic regulation of nucleosome positioning in the human genome. Cell.

[23] Boyle AP, Davis S, Shulha HP, Meltzer P, Margulies EH, Weng Z, Furey TS, Crawford GE (2008). High-resolution mapping and characterization of open chromatin across the genome. Cell.

[24] Giresi PG, Kim J, McDaniell RM, Iyer VR, Lieb JD (2007). FAIRE (Formaldehyde-Assisted Isolation of Regulatory Elements) isolates active regulatory elements from human chromatin. Genome Res.

[25] Waki H, Nakamura M, Yamauchi T, Wakabayashi K, Yu J, Hirose-Yotsuya L, Take K, Sun W, Iwabu M, Okada-Iwabu M, Fujita T, Aoyama T, Tsutsumi S, Ueki K, Kodama T, Sakai J, Aburatani H, Kadowaki T (2011). Global mapping of cell type-specific open chromatin by FAIRE-seq reveals the regulatory role of the NFI family in adipocyte differentiation. PLoS Genet.

[26] Yuan G-C, Liu Y-J, Dion MF, Slack MD, Wu LF, Altschuler SJ, Rando OJ (2005). Genome-scale identification of nucleosome positions in S. cerevisiae. Science.

[27] Henikoff JG, Belsky JA, Krassovsky K, MacAlpine DM, Henikoff S (2011). Epigenome characterization at single base-pair resolution. Proc Natl Acad Sci U S A.

[28] Kent NA, Adams S, Moorhouse A, Paszkiewicz K (2011). Chromatin particle spectrum analysis: a method for comparative chromatin structure analysis using paired-end mode next-generation DNA sequencing. Nucleic Acids Res.

[29] Stamatoyannopoulos JA, Snyder M, Hardison R, Ren B, Gingeras T, Gilbert DM, Groudine M, Bender M, Kaul R, Canfield T, Giste E, Johnson A, Zhang M, Balasundaram G, Byron R, Roach V, Sabo PJ, Sandstrom R, Stehling AS, Thurman RE, Weissman SM, Cayting P, Hariharan M, Lian J, Cheng Y, Landt SG, Ma Z, Wold BJ, Dekker J, Mouse ENCODE Consortium (2012). An encyclopedia of mouse DNA elements (Mouse ENCODE). Genome Biol.

[30] Liberator PA, Lingrel JB (1984). Restriction endonuclease accessibility of the developmentally regulated goat gamma-, beta C-, and beta A-globin genes in chromatin. Differences in 5’ regions which show unusually high sequence homology. J Biol Chem.

[31] Almer A, Hörz W (1986). Nuclease hypersensitive regions with adjacent positioned nucleosomes mark the gene boundaries of the PHO5/PHO3 locus in yeast. EMBO J.

[32] Logie C, Peterson CL (1997). Catalytic activity of the yeast SWI/SNF complex on reconstituted nucleosome arrays. EMBO J.

[33] Narlikar GJ, Phelan ML, Kingston RE (2001). Generation and interconversion of multiple distinct nucleosomal states as a mechanism for catalyzing chromatin fluidity. Mol Cell.

[34] Ohkawa Y, Marfella CGA, Imbalzano AN (2006). Skeletal muscle specification by myogenin and Mef2D via the SWI/SNF ATPase Brg1. EMBO J.

[35] Gargiulo G, Levy S, Bucci G, Romanenghi M, Fornasari L, Beeson KY, Goldberg SM, Cesaroni M, Ballarini M, Santoro F, Bezman N, Frigè G, Gregory PD, Holmes MC, Strausberg RL, Pelicci PG, Urnov FD, Minucci S (2009). NA-Seq: a discovery tool for the analysis of chromatin structure and dynamics during differentiation. Dev Cell.

[36] Pfeiffer W, Zachau HG (1980). Accessibility of expressed and non-expressed genes to a restriction nuclease. Nucleic Acids Res.

[37] Felsenfeld G (1992). Chromatin as an essential part of the transcriptional mechanism. Nature.

[38] Kornberg RD, Lorch Y (1992). Chromatin structure and transcription. Annu Rev Cell Biol.

[39] Pearson JC, Lemons D, McGinnis W (2005). Modulating Hox gene functions during animal body patterning. Nat Rev Genet.

[40] Davie JR, Saunders CA (1981). Chemical composition of nucleosomes among domains of calf thymus chromatin differing in micrococcal nuclease accessibility and solubility properties. J Biol Chem.

[41] Xi H, Shulha HP, Lin JM, Vales TR, Fu Y, Bodine DM, McKay RDG, Chenoweth JG, Tesar PJ, Furey TS, Ren B, Weng Z, Crawford GE (2007). Identification and characterization of cell type-specific and ubiquitous chromatin regulatory structures in the human genome. PLoS Genet.

[42] Saeed S, Logie C, Francoijs K-J, Frigè G, Romanenghi M, Nielsen FG, Raats L, Shahhoseini M, Huynen M, Altucci L, Minucci S, Martens JHA, Stunnenberg HG (2012). Chromatin accessibility, p300, and histone acetylation define PML-RARα and AML1-ETO binding sites in acute myeloid leukemia. Blood.

[43] Carone BR, Hung J-H, Hainer SJ, Chou M-T, Carone DM, Weng Z, Fazzio TG, Rando OJ (2014). High-resolution mapping of chromatin packaging in mouse embryonic stem cells and sperm. Dev Cell.

[44] Fedoriw AM, Stein P, Svoboda P, Schultz RM, Bartolomei MS (2004). Transgenic RNAi reveals essential function for CTCF in H19 gene imprinting. Science.

[45] Szabó PE, Tang S-HE, Silva FJ, Tsark WMK, Mann JR (2004). Role of CTCF binding sites in the Igf2/H19 imprinting control region. Mol Cell Biol.

[46] Kurukuti S, Tiwari VK, Tavoosidana G, Pugacheva E, Murrell A, Zhao Z, Lobanenkov V, Reik W, Ohlsson R (2006). CTCF binding at the H19 imprinting control region mediates maternally inherited higher-order chromatin conformation to restrict enhancer access to Igf2. Proc Natl Acad Sci U S A.

[47] Fu Y, Sinha M, Peterson CL, Weng Z (2008). The insulator binding protein CTCF positions 20 nucleosomes around its binding sites across the human genome. PLoS Genet.

[48] Cuddapah S, Jothi R, Schones DE, Roh T-Y, Cui K, Zhao K (2009). Global analysis of the insulator binding protein CTCF in chromatin barrier regions reveals demarcation of active and repressive domains. Genome Res.

[49] Cao K, Lailler N, Zhang Y, Kumar A, Uppal K, Liu Z, Lee EK, Wu H, Medrzycki M, Pan C, Ho P-Y, Cooper GP, Dong X, Bock C, Bouhassira EE, Fan Y (2013). High-resolution mapping of h1 linker histone variants in embryonic stem cells. PLoS Genet.

[50] Niwa H, Miyazaki J, Smith AG (2000). Quantitative expression of Oct-3/4 defines differentiation, dedifferentiation or self-renewal of ES cells. Nat Genet.

[51] Skene PJ, Henikoff S (2013). Histone variants in pluripotency and disease. Development.

[52] Hu G, Cui K, Northrup D, Liu C, Wang C, Tang Q, Ge K, Levens D, Crane-Robinson C, Zhao K (2013). H2A.Z facilitates access of active and repressive complexes to chromatin in embryonic stem cell self-renewal and differentiation. Cell Stem Cell.

[53] Creyghton MP, Markoulaki S, Levine SS, Hanna J, Lodato MA, Sha K, Young RA, Jaenisch R, Boyer LA (2008). H2A.Z is enriched at polycomb complex target genes in ES cells and is necessary for lineage commitment. Cell.

[54] Ahmad K, Henikoff S (2002). The histone variant H3.3 marks active chromatin by replication-independent nucleosome assembly. Mol Cell.

[55] Goldberg AD, Banaszynski LA, Noh K-M, Lewis PW, Elsaesser SJ, Stadler S, Dewell S, Law M, Guo X, Li X, Wen D, Chapgier A, DeKelver RC, Miller JC, Lee Y-L, Boydston EA, Holmes MC, Gregory PD, Greally JM, Rafii S, Yang C, Scambler PJ, Garrick D, Gibbons RJ, Higgs DR, Cristea IM, Urnov FD, Zheng D, Allis CD (2010). Distinct factors control histone variant H3.3 localization at specific genomic regions. Cell.

[56] Szenker E, Ray-Gallet D, Almouzni G (2011). The double face of the histone variant H3.3. Cell Res.

[57] Mizuguchi G, Shen X, Landry J, Wu W-H, Sen S, Wu C (2004). ATP-driven exchange of histone H2AZ variant catalyzed by SWR1 chromatin remodeling complex. Science.

[58] Tagami H, Ray-Gallet D, Almouzni G, Nakatani Y (2004). Histone H3.1 and H3.3 complexes mediate nucleosome assembly pathways dependent or independent of DNA synthesis. Cell.

[59] Miller MR, Dunham JP, Amores A, Cresko WA, Johnson EA (2007). Rapid and cost-effective polymorphism identification and genotyping using restriction site associated DNA (RAD) markers. Genome Res.

[60] Baird NA, Etter PD, Atwood TS, Currey MC, Shiver AL, Lewis ZA, Selker EU, Cresko WA, Johnson EA (2008). Rapid SNP discovery and genetic mapping using sequenced RAD markers. PLoS ONE.

[61] McClelland M (1981). The effect of sequence specific DNA methylation on restriction endonuclease cleavage. Nucleic Acids Res.

[62] Doetschman T, Gregg RG, Maeda N, Hooper ML, Melton DW, Thompson S, Smithies O (1987). Targetted correction of a mutant HPRT gene in mouse embryonic stem cells. Nature.

[63] Yildirim O, Li R, Hung J-H, Chen PB, Dong X, Ee L-S, Weng Z, Rando OJ, Fazzio TG (2011). Mbd3/NURD complex regulates expression of 5-hydroxymethylcytosine marked genes in embryonic stem cells. Cell.

[64] Langmead B, Trapnell C, Pop M, Salzberg SL (2009). Ultrafast and memory-efficient alignment of short DNA sequences to the human genome. Genome Biol.

[65] Zhu LJ, Gazin C, Lawson ND, Pagès H, Lin SM, Lapointe DS, Green MR (2010). ChIPpeakAnno: a Bioconductor package to annotate ChIP-seq and ChIP-chip data. BMC Bioinformatics.

[66] Heinz S, Benner C, Spann N, Bertolino E, Lin YC, Laslo P, Cheng JX, Murre C, Singh H, Glass CK (2010). Simple combinations of lineage-determining transcription factors prime cis-regulatory elements required for macrophage and B cell identities. Mol Cell.

[67] Yang D, Buchholz F, Huang Z, Goga A, Chen C-Y, Brodsky FM, Bishop JM (2002). Short RNA duplexes produced by hydrolysis with Escherichia coli RNase III mediate effective RNA interference in mammalian cells. Proc Natl Acad Sci U S A.

[68] Fazzio TG, Huff JT, Panning B (2008). An RNAi screen of chromatin proteins identifies Tip60-p400 as a regulator of embryonic stem cell identity. Cell.

[69] Chen PB, Hung J-H, Hickman TL, Coles AH, Carey JF, Weng Z, Chu F, Fazzio TG (2013). Hdac6 regulates Tip60-p400 function in stem cells. Elife.

