# Supplementary figures and images for: Unbiased chromatin accessibility profiling by RED-seq uncovers unique features of nucleosome variants in vivo
Source: BMC Genomics. 2014 Dec 15;15(1):1104. doi: 10.1186/1471-2164-15-1104 (PMC4378318; doi:10.1186/1471-2164-15-1104)

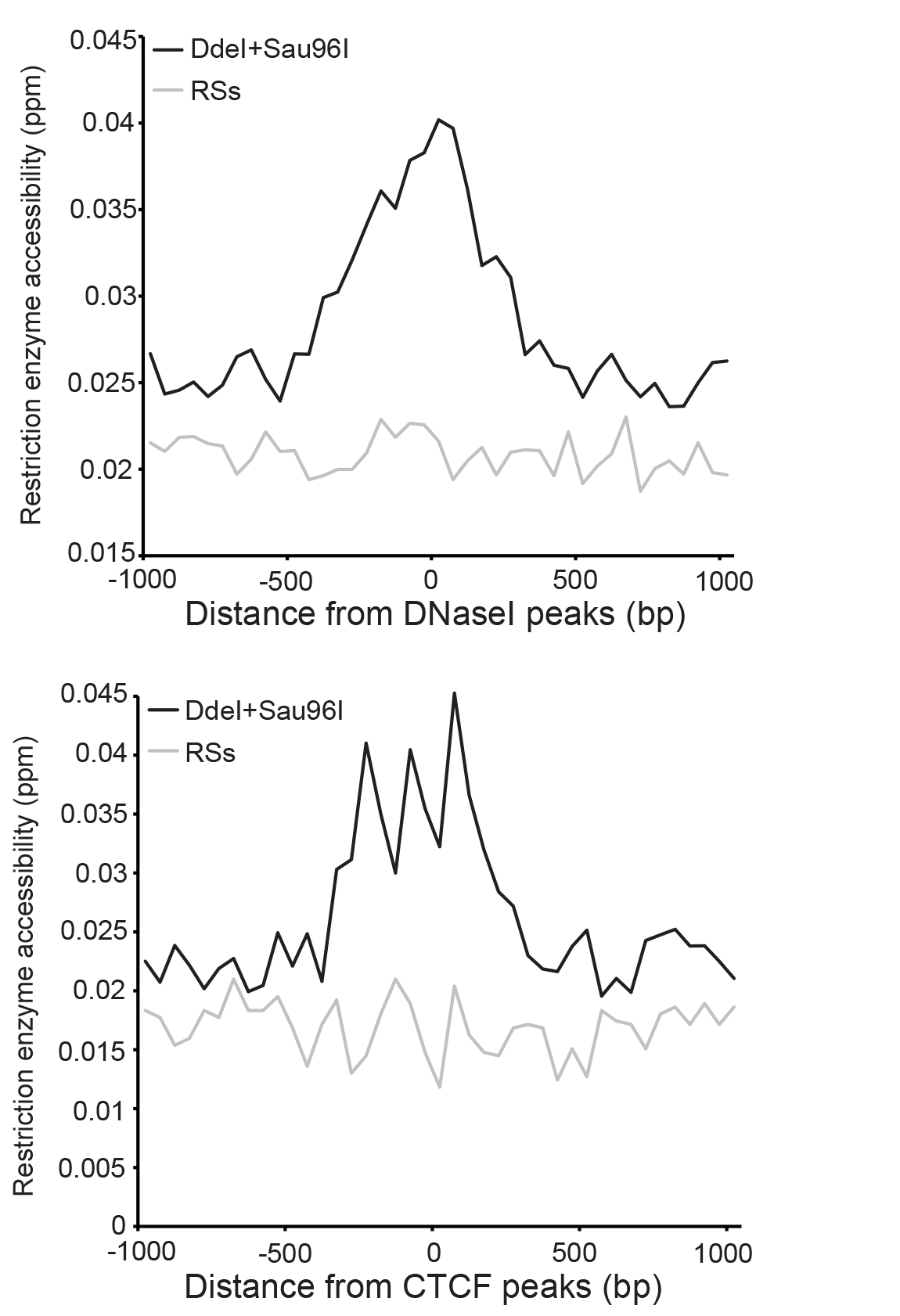

Supplement: Supplementary file 1 — Additional file 1: Testing RED-seq using two REs. Average RE accessibility within a 2 kb region (-1000 to +1000 bp from the peaks) of DHSs or CTCF binding sites as measured by RED-seq using Sau96I and DdeI, plotted as in Figure 3. (TIFF 221 KB) [file 12864_2014_6869_MOESM1_ESM.tiff]

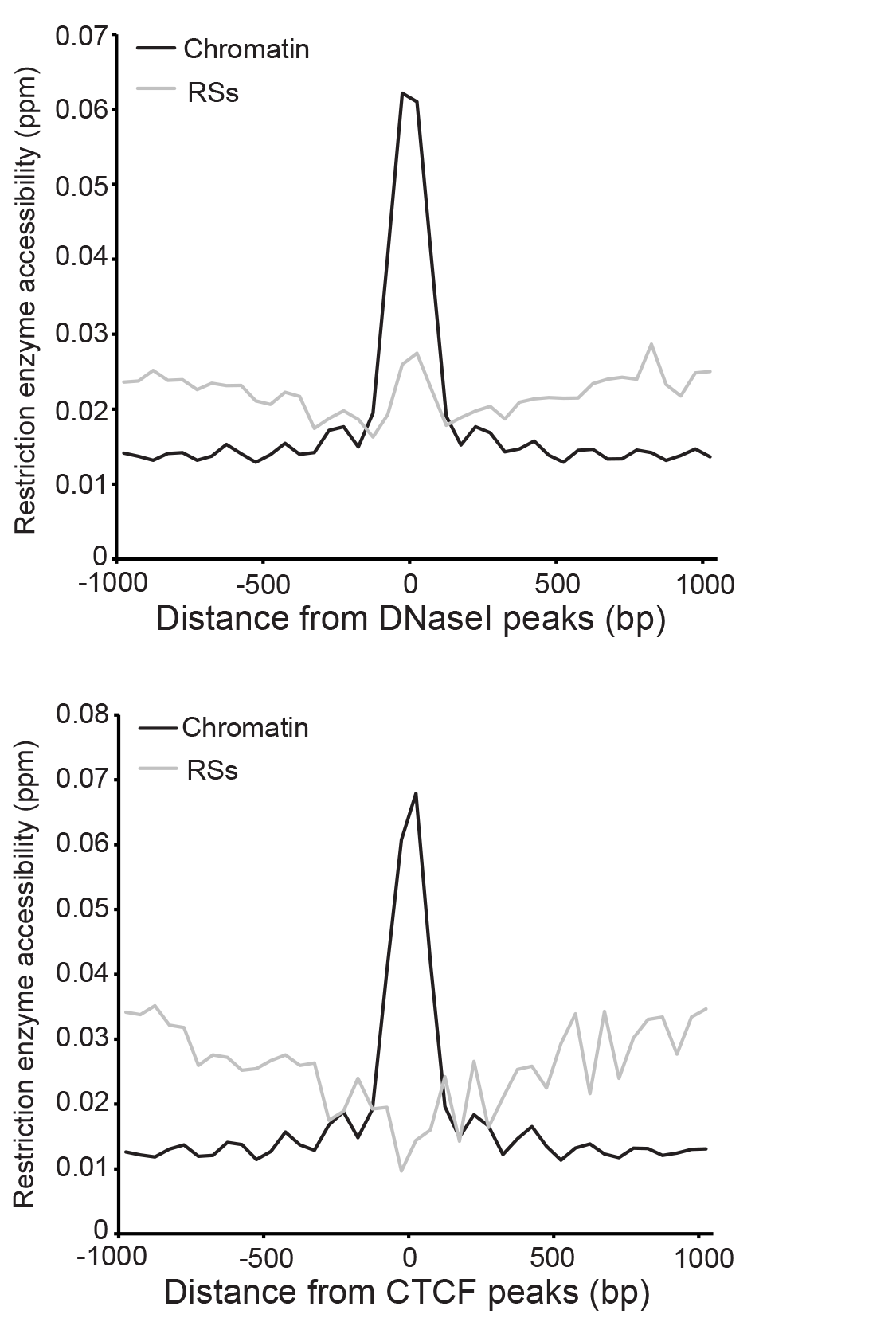

Supplement: Supplementary file 2 — Additional file 2: Re-analysis of NA-seq data. Average RE accessibility within a 2 kb region (-1000 to +1000 bp from the peaks) of DHSs or CTCF binding sites from published NA-seq data [GEO:GSE30254], plotted as in Figure 3. (TIFF 205 KB) [file 12864_2014_6869_MOESM2_ESM.tiff]
