# Supplementary material for: Unbiased chromatin accessibility profiling by RED-seq uncovers unique features of nucleosome variants in vivo
Source: BMC Genomics. 2014 Dec 15;15(1):1104. doi: 10.1186/1471-2164-15-1104 (PMC4378318; doi:10.1186/1471-2164-15-1104)
Supplement: Supplementary file 3 — Additional file 3: Sequences of barcoded and biotinylated adaptors. (PDF 42 KB) [file 12864_2014_6869_MOESM3_ESM.pdf]

Additional File 3. Sequences of barcoded and biotinylated adaptors.

Name	Forward sequence	Reverse sequence
Modified-Biotin-Set2	p-GCAGATCGGAAGAGCGTCGTGTAGGGAAAGAGTGT	Biotin-ACACTCTTTCCCTACACGACGCTCTTCCGATCTGCT
Modified-Set2	p-GCAGATCGGAAGAGCTCGTATGCCGTCTTCTGCTTG	CAAGCAGAAGACGGCATACGAGCTCTTCCGATCTGCT
Modified-Biotin-Set5	p-CGTAGATCGGAAGAGCGTCGTGTAGGGAAAGAGTGT	Biotin-ACACTCTTTCCCTACACGACGCTCTTCCGATCTACGT
Modified-Set5	p-CGTAGATCGGAAGAGCTCGTATGCCGTCTTCTGCTTG	CAAGCAGAAGACGGCATACGAGCTCTTCCGATCTACGT
Modified-Biotin-Set6	p-ATGAGATCGGAAGAGCGTCGTGTAGGGAAAGAGTGT	Biotin-ACACTCTTTCCCTACACGACGCTCTTCCGATCTCATT
Modified-Set6	p-ATGAGATCGGAAGAGCTCGTATGCCGTCTTCTGCTTG	CAAGCAGAAGACGGCATACGAGCTCTTCCGATCTCATT
Modified-Biotin-Set8	p-GCTAGATCGGAAGAGCGTCGTGTAGGGAAAGAGTGT	Biotin-ACACTCTTTCCCTACACGACGCTCTTCCGATCTAGCT
Modified-Set8	p-GCTAGATCGGAAGAGCGGTTCAGCAGGAATGCCGAG	CTCGGCATTCCTGCTGAACCGCTCTTCCGATCTAGCT
Modified-Biotin-Set10	p-CGAAGATCGGAAGAGCGTCGTGTAGGGAAAGAGTGT	Biotin-ACACTCTTTCCCTACACGACGCTCTTCCGATCTTCGT
Modified-Set10	p-CGAAGATCGGAAGAGCGGTTCAGCAGGAATGCCGAG	CTCGGCATTCCTGCTGAACCGCTCTTCCGATCTTCG
