# Supplementary material for: Unbiased chromatin accessibility profiling by RED-seq uncovers unique features of nucleosome variants in vivo
Source: BMC Genomics. 2014 Dec 15;15(1):1104. doi: 10.1186/1471-2164-15-1104 (PMC4378318; doi:10.1186/1471-2164-15-1104)
Supplement: Supplementary file 4 — Additional file 4: Sequences of qPCR primers for DHSs. (PDF 40 KB) [file 12864_2014_6869_MOESM4_ESM.pdf]

Additional File 4. qPCR primers for DHSs.

Name	Forward sequence	Reverse sequence
DHS-Chr1	CAGGTGGAGGGAGACAGAGA	TGGCAAGAAGGACTGTTGGT
DHS-Chr6	TGCTCTCTTCCACCCTCTGT	GGAGAGCCCTAGACTGGAAC
DHS-Chr10	CAGTGCCCAGACTCATCCTG	TGATGTCACATGCCTAGCCA
DHS-Chr11	TACCTGGAACCGGTTTACCC	CCTGAGCATCGACTCTCCAA
DHS-Chr12	GGTTGGAGTTGGAATCCGCA	AGCAAAGAAGGCACAGCTATG
DHS-Chr16	GCATGCCTCGTACCTGTGTA	GCAGGTAGGCGCTCTTACAG
