# Supplementary material for: Unbiased chromatin accessibility profiling by RED-seq uncovers unique features of nucleosome variants in vivo
Source: BMC Genomics. 2014 Dec 15;15(1):1104. doi: 10.1186/1471-2164-15-1104 (PMC4378318; doi:10.1186/1471-2164-15-1104)
Supplement: Supplementary file 5 — Additional file 5: Sequences of qPCR primers for CTCF binding sites. (PDF 48 KB) [file 12864_2014_6869_MOESM5_ESM.pdf]

Additional File 5. qPCR primers for CTCF binding sites.

Name	Forward sequence	Reverse sequence
CTCF-Chr2	GGTTACCAAACCAGGTAAGATTTG	CTGCTCAGAAGAAGAAACTGGA
CTCF-Chr16	CATGTATGCCTACTTGCCAGA	TGCTCTTACAGAGGTCCTGA
CTCF-Chr15	CACCCACATGGCAGCTAATA	CTGCTTGTGTGTGCACTTTATG
CTCF-Chr4	ACCACTGACTGCTGAAAGTT	CTGTTAGAAGGACTGACTGGTG
CTCF-Chr1-1	ACTATGCATGCAGTACCTGTG	TGGCTCACGACTGTCTCTAA
CTCF-Chr1-2	GTACATGTAGTGCTCACAGAGG	CAATGGCTGCTCTTCCAGTA
CTCF-Chr7	TGCATCATGTGTGGACCTAAT	CAATTCCCAGAGCCCATGTA
CTCF-control1	GGTCCTGAACCCTTGAGAGA	GCCCTCTAGTGGCAAAGAAA
CTCF-control2	GGCGATGAACTTTACCCATC	TGTTTGCTATAAAACAGGACCAGA
